# In Vivo Evaluation of a New Recombinant Hyaluronidase to Improve Gene Electro-Transfer Protocols for DNA-Based Drug Delivery against Cancer

**DOI:** 10.3390/cancers10110405

**Published:** 2018-10-28

**Authors:** Mariangela De Robertis, Lise Pasquet, Luisa Loiacono, Elisabeth Bellard, Luciano Messina, Susanna Vaccaro, Roberta Di Pasquale, Vito Michele Fazio, Marie-Pierre Rols, Justin Teissie, Muriel Golzio, Emanuela Signori

**Affiliations:** 1 Department of Biosciences, Biotechnology and Biopharmaceutics, University of Bari “A. Moro”, via Orabona 4, 70126 Bari, Italy; mariangela.derobertis@gmail.com; 2 Laboratory of Molecular Medicine and Biotechnology, University Campus Bio-Medico of Rome, via Alvaro del Portillo 21, 00128 Rome, Italy; luisa.loiacono@gmail.com (L.L.); FAZIO@unicampus.it (V.M.F.); 3 CNR―Institute of Translational Pharmacology, Via Fosso del Cavaliere 100, 00133 Rome, Italy; 4 Vaccine Branch, CCR, NCI, NIH, Bethesda, MD 20892, USA; lisy.pasquet@gmail.com; 5 Institut de Pharmacologie et Biologie Structurale, IPBS, Université de Toulouse, CNRS, UPS, BP64182, 205 Route de Narbonne, 31077 Toulouse, France; Elisabeth.Bellard@ipbs.fr (E.B.); Marie-Pierre.Rols@ipbs.fr (M.-P.R.); 6 New Drug Modalities, Drug Safety and Metabolism, IMED Biotech Unit, AstraZeneca, Cambridge CB4 0WG, UK; 7 Fidia Farmaceutici S.p.A., Local Unit Fidia Research Sud, Contrada Pizzuta snc, 96017 Noto, Siracusa, Italy; LMessina@fidiapharma.it (L.M.); SVaccaro@fidiapharma.it (S.V.); RDiPasquale@fidiapharma.it (R.D.P.); 8 Fondazione IRCCS Casa Sollievo della Sofferenza, Laboratorio di Oncologia, viale dei Cappuccini, 71013 San Giovanni Rotondo (FG), Italy

**Keywords:** DNA-based drug delivery, hyaluronidase, gene electro-transfer, DNA vaccination, cancer therapy, oncoimmunology, translational protocols

## Abstract

Cancer vaccines based on plasmid DNA represent a good therapeutic perspective, despite their low potency. Animal-derived hyaluronidases (Hyals) are employed in oncological clinical practice. Hyal has been also demonstrated to be a good enhancer of intramuscular Gene Electro-Transfer (GET) efficiency in anti-cancer preclinical protocols, with increased transfected cells and higher expression of the encoded genes. Nevertheless, the use of animal-derived Hyals results limited respect to their potentialities, since such preparations could be affected by low purity, variable potency and uncertain safety. To improve the delivery of intramuscular GET-based protocols in mouse, we investigated a new recombinant Hyal, the rHyal-*sk*, to assess in vivo safety and activity of this treatment at cellular and biochemical levels. We evaluated the cellular events and the inflammation chemical mediators involved at different time points after rHyal-*sk* administration plus GET. Our results demonstrated the in vivo safety and efficacy of rHyal-*sk* when injected once intramuscularly in association with GET, with no toxicity, good plasmid in-take ability, useful inflammatory response activation, and low immunogenicity. Following these findings, we would recommend the use of the new rHyal-*sk* for the delivery of DNA-based vaccines and immunotherapy, as well as into clinical practice, for tumor disease treatments.

## 1. Introduction

Cancer vaccines based on plasmid DNA have been proved as good alternative to the more traditional subunit vaccines like recombinant proteins and peptides. Nevertheless, their limited potency is one of the “core” issues of DNA vaccination [1]. 

To increase the efficacy of genetic vaccines, several studies are focused on delivery systems. They envisage compounds and technical strategies helping to deliver the DNA vaccines into the right cell compartment for obtaining maximum potency together with improved safety profile. 

Hyaluronidases (Hyals) or hyaluronoglucosaminidases are a family of enzymes degrading hyaluronic acid (HA), also called hyaluronan, which is an important component of the extracellular matrix (ECM) of the connective and hepitelial tissues. Hyals hydrolyze the hexosaminidic β (1–4) linkages between *N*-acetyl-*D*-glucosamine and *D*-glucuronic acid residues in HA molecule and release HA fragments [2]. The vertebrate Hyals generate a various range of HA oligomers [3]. Some bacteria, such as *Staphylococcus aureus*, *Streptococcus pyogenes*, and *Clostridium perfringens*, produce Hyals as a virulence factor, that facilitates toxins diffusion and bacterial motility through the host’s tissues [4,5]. It is also well known that HA and HA-binding proteins play a role in inflammation, tissue injury and repair through regulating inflammatory cell recruitment, release of inflammatory cytokines and cell migration [6]. 

Hyal is proven to greatly increase the dispersion and absorption of subcutaneously injected large molecules. It is used as an adjunct to nonpharmacological management of extravasation of selected antineoplastic agents, and in the management of extravasation contrast media [7,8]. Bacterial [9], Bovine (Amphadase^TM^, Hydase^TM^) [10,11], ovine (Vitrase^TM^) [12], and human recombinant (Hylenex^TM^) [13] Hyal preparations have been developed over the years for wider uses in the clinical practice.

In the years 1940–1950, Hyal became popular in the vaccination field as a “spreading factor” used for a better diffusion of traditional vaccines at the site of injection [14,15,16]. Recently, the positive effects of Hyal were shown in gene therapy targeting neurons [17], and in pre-clinical protocols based on Gene Electro-Transfer (GET) [18,19,20,21]. 

GET is a method that allows the targeted transfer of plasmid DNA into cells in a tissue and promotes efficient gene expression. It results from the direct application of electric field pulses, avoiding the use of viral vectors. The application of controlled electric pulses induces a transient permeabilisation of the plasma membrane and the uptake of the negatively charged plasmid DNA inside the cell [22,23]. GET has been applied in vivo since 1998 [24] in different tissues and organs [25,26,27]. During the last decade, several clinical trials based on GET were initiated [28]. Many of them are focused in developing anti-tumor vaccine therapy. In a phase I clinical trial on patients with metastatic melanoma, 10% of the patients treated by GET of a plasmid encoding the cytokine IL-12 exhibited a complete regression of all metastases (treated and untreated) while 42% displayed a stable disease or partial regression [29].

The strength of GET is to induce a massive immune response. The transfected plasmid encoding specific antigens is uptaken by muscular cells and by professional antigen-presenting-cells (APC) present in the pulsed tissue. These APC migrate to the draining lymph nodes where they activate the adaptive immune response through stimulation of CD4+ and CD8+ T cells. Moreover, the delivered electric field pulse by itself induces a local inflammation characterized by the attraction of macrophages and dendritic cells amplifying the strength and the duration of the immune response [30,31,32]. However, in cancer, because of the self-origin of the cancer cells, only few antigens associated to the tumors are efficiently immunogenic (i.e., able to trigger an effective immune response). Therefore, the development of a GET-based therapy able to improve the response of the immune system is still needed [33].

It is known that the treatment of skeletal muscle with Hyal allows the application of lower levels of electric fields, thus reducing muscle fibres damage [34]. Moreover, in a recent article on the effect of Hyal on mouse skeletal muscle, pretreatment with this enzyme was demonstrated to be a good enhancer of GET efficiency in gene vaccination protocols. In particular, when used in combination with pulsed electric fields, Hyal increased the release of pro-inflammatory cytokines (Interleukin-1β (IL-1β), tumor necrosis factor (TNF-α), Interleukin-6 (IL-6)) and favoured local APC infiltration in a defined time-window between day 4 and day 7 following cytokine induction [21]. The final effect was an improved delivery of the DNA vaccine and a general enhancement in terms of treatment efficacy. These observations underline the relevance of appropriate technologies and molecules able to improve the drug delivery in GET protocols to be introduced into the clinical practice.

Bovine-derived Hyal (Amphadase^TM^) [10] and ovine-derived Hyal (Vitrase^TM^) [12] were approved by the Food and Drug Administration (FDA) in 2004; a second bovine-derived Hyal (Hydase^TM^) [11] was subsequently approved in 2005. Notwithstanding that animal-derived Hyals are regularly employed, the production of such formulations have been generally reported to be limited due to low purity, variable potency and uncertain safety. These preparations are often contaminated with proteases, immunoglobulins and other factors that increase capillary permeability and may induce IgE-mediated allergic reactions when repeatedly administrated, generally precluding their subsequent use. Importantly, many case reports of adverse reactions were published in medical journals connected with the use of animal-derived Hyals that were subsequently taken off the market [35,36,37,38]. Therefore, they appear to bring a risk for application in human patients and also preclinical protocols based on DNA vaccination by pretreatment with animal-derived Hyal and electroporation could be narrowly proposed in clinical trials. 

A human recombinant Hyal (Hylenex^TM^, Halozyme Therapeutics Inc., San Diego, CA, USA) has been produced by genetically engineered Chinese Hamster Ovary (CHO) cells containing a DNA plasmid encoding for a soluble fragment of human Hyal (PH20) [13], and has been recently approved by the FDA for use as an adjuvant agent in subcutaneous fluid administration for achieving hydration, to increase the dispersion and absorption of other injected drugs and as an adjunct in subcutaneous urography for improving resorption of radiopaque agents [39]. This product has been widely used in man, however no data were available describing its use for GET assays. However, a possible utilization of recombinant human PH20 Hyal (rHuPH20) in GET protocols, i.e., in the context of a high stimulation of the immune response, could have a limitation related to the eventual production of non-neutralizing antibodies against rHuPH20, with associated risk of cross-reactivity versus endogenous PH20 with potential long-term effects [40,41].

A novel Hyal, called rHyal-*sk* (Fidia Farmaceutici S.p.A, Abano Terme, Padova, Italy) was produced as a recombinant protein in a non-pathogenic bacteria (GRAS product: Generally Regarded As Safe) and obtained by a new protocol of extraction [9,42]. rHyal-*sk* is a non-glycosylated, cysteine disulfide bond free enzyme containing a novel bacterial catalytic domain with high enzymatic activity and it is characterized by a very low molecular weight (about 22 kDa), an excellent purity profile, high stability to proteolytic enzymes, at low/high pH and at high temperatures up to 70 °C. Additional properties include: long shelf-life, high performance at physiological pH and at body temperature, and it is not inhibited by human blood [9,42]. In addition, rHyal-*sk* exhibits remarkable substrate specificity for HA [43] and no risk of animal cross-infection.

In order to improve GET-based protocols of therapeutic plasmid injection and move rapidly to safe and efficient translational gene therapy protocols, we evaluated the efficacy and safety of this novel Hyal. Here we describe a pretreatment of murine skeletal muscle with rHyal-*sk* followed by GET of plasmid DNA (coding for the fluorescent protein tdTomato) to improve the transfection efficiency of plasmids within the injected muscle. Results were compared with a pretreatment with bHyal, already known to bring a positive effect on electrotransfection [18,21,44]. The evaluation of this new kind of hyaluronidase was performed both in terms of the overall levels of gene expression in the transfected muscle fibres by fluorescence imaging and morphological damage occurring in the muscle. We also investigated the potential to activate a local proinflammatory immune response in injected muscle, a crucial aspect that should be considered in the optimization of GET protocols against cancer. 

## 2. Results

### 2.1. Principal Features of rHyal-sk

Cloning, recombinant protein expression and final purification of rHyal-*sk* were successfully performed and optimized in BL21 Escherichia coli. The purification process resulted in recombinant bacterial Hyal with a ≥99% purity and a specific activity of ≥40,000 U/mg [42].

rHyal-*sk* showed the same effectiveness as native Hyal, but with a considerably better safety and purity profile [9,42], including no risk of animal cross-infection as compared to available options. Through several preliminary biochemical studies against available options we demonstrated that rHyal-*sk* display many advantageous features, such as superior activity at physiological pH and better stability at physiological temperature than the available products, stability up to 70 °C, higher stability against proteolityc enzymes, higher resistance to human serum [9,42]. 

The toxicity study and the evaluation of the neutralizing ADA (nADA) induction demonstrated that no toxicological findings were recorded in any of the subcutaneously treated rats. There was neither premature death nor rHyal-*sk*-related gross pathology findings or organ weight changes nor macroscopic changes. Microscopic findings were observed only in animals treated at 10,500 U/kg and consisted of minor alterations. No alteration was observed after the recovery period. However, there was a clear indication of an immunogenic potential of rHyal-*sk*, when administered subcutaneously for a period of 4 weeks with a 2 weeks recovery in rats, as shown in animals from treated groups with increasing doses of rHyal-*sk* (groups 2 (6/6 animals), 3 (5/6 animals), and group 4 (2/6 animals) (Table 1). In addition, all samples which were confirmed positive for the presence of ADA were further investigated in the nAbs assay, where we obtained that the production of nADA was present at all dose levels, although not in all animals and not in a dose-related way (data not shown). 

### 2.2. rHyal-sk Plus GET Ensures High Gene Electrotransfection Efficacy

Four mice skeletal muscles were treated with the rHyal-*sk*. Two hours later, muscles were electrotransfected with a plasmid encoding the fluorescent protein tdTomato used as a reporter gene of electrotransfection efficacy. Reference control muscles (n = 4) were treated with the bHyal. Control muscles were injected with the Hyal alone (n = 4) or with LPS (n = 4) as positive control of inflammation. Negative controls consisted of untreated muscles (n = 4).

**Figure 1:**
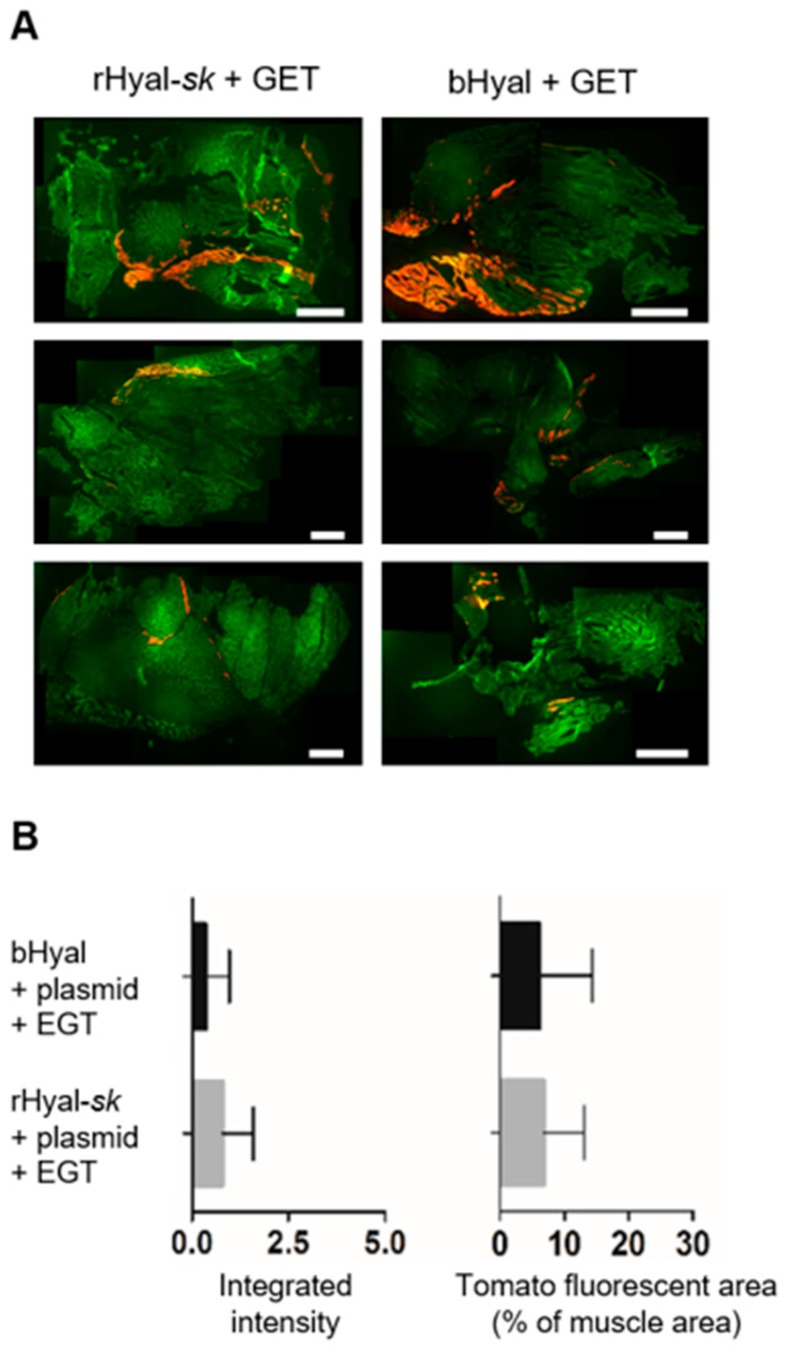
Transfection efficiency of the combination plasmid GET plus rHyal-*sk*. (**A**) Representative cryosections of three skeletal muscles harvested 7 days after tdTomato plasmid GET in presence of rHyal-*sk* or bHyal. Fluorescence images (Red: tdTomato, Green: tissue autofluorescence) were acquired on a Macroscope after mounting. 5× magnification. Scale bars, 1 mm. (**B**) Fluorescence integrated intensity and area of expression of the tdTomato protein were measured in presence of rHyal-*sk* (n = 4 muscles) or bHyal (n = 4 muscles). Data are Mean ± SD, *p =* 0.63 and 0.95 respectively (n.s.). Statistical analysis: Mann Whitney test.

To visually quantify the efficacy of the plasmid electrotransfection coupled to rHyal-*sk* treatment, we compared the fluorescence intensity and expression area of the tdTomato reporter gene between muscles treated with plasmid tdTomato by GET in association with rHyal-*sk* (n = 4) or bHyal (n = 4) (Figure 1) in muscles collected 7 days after treatment. As a good efficiency result, no statistical difference was observed in the intensity or in the area of expression (Figure 1) of the tdTomato protein whether muscles were treated with rHyal-*sk* or bHyal. The area of tdTomato expression was heterogenous between different muscles wathever the hyaluronidase used, whereas the intensity expression was similar.

### 2.3. rHyal-sk Plus GET induces only a Transient Alteration of Muscle Morphology after Pulse Delivery

To evaluate the effect on the tissue damage and recruitment of monocytes and granulocytes infiltration in mouse skeletal muscle after rHyal-*sk* pretreatment and application of electrical field pulses, we examined skeletal muscle morphology looking for the presence of infiltrating leukocytes at three different time points: 3 h (d0), 7 days and 14 days. We tested the effect of rHyal-*sk* after the following treatments: Hyal injection alone (rHyal-*sk*) (n = 4) or in combination with GET (rHyal-*sk* plus GET) (n = 4). Muscles injected with bHyal alone (bHyal) (n = 4) or in combination with GET (bHyal plus GET) (n = 4) were also tested as reference samples. Skeletal muscles collected at the same times (0, 7 and 14 days) after LPS injection were considered as the positive control. Previous experiments with GET without hyaluronidase under the same experimental condition widely demonstrated the positive contribution of a pre-treatment with bHyal as reported [18,21,44].

**Figure 2:**
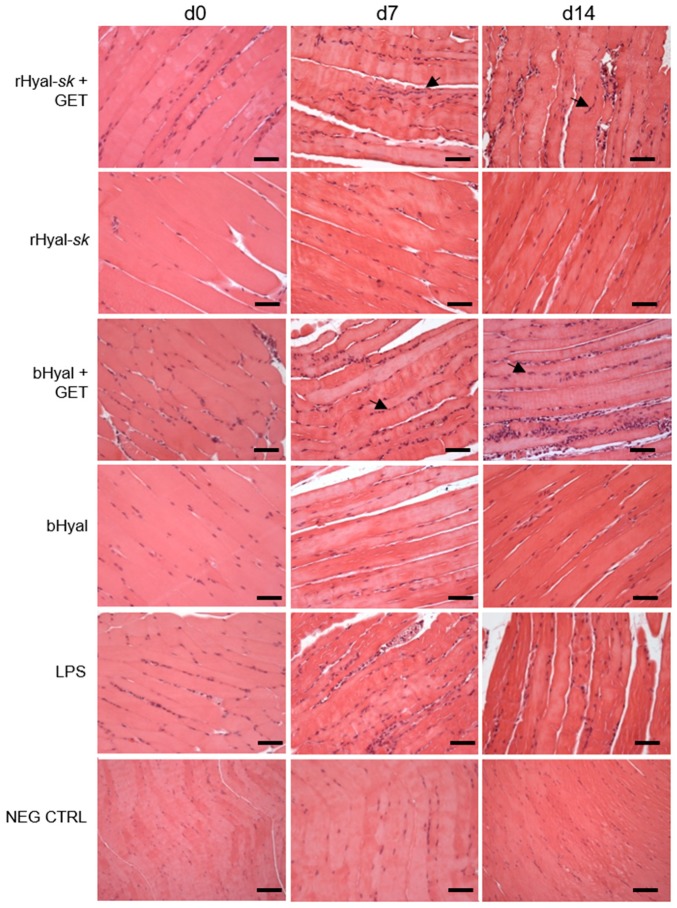
Histological analysis of the morphology and the regenerative process of skeletal muscle after treatment with rHyal-*sk* in the presence and absence of GET. Paraffin sections of skeletal muscles taken 3 h (d0), d7 and d14 after treatment with rHyal-*sk* plus GET or rHyal-*sk* alone are shown. bHyal plus GET or bHyal alone were used as reference muscles. Positive control: muscles injected with LPS. Negative control: untreated muscle. Arrows indicate nuclei of regenerating muscle fibers. 20× magnification. Scale bars, 50 μm. rHyal-*sk*, recombinant Hyal from Fidia Farmaceutici; bHyal, bovine Hyal from Sigma-Aldrich (St. Louis, MO, USA); Hyal + GET, electrotransfer after Hyal injection; LPS, lipopolysaccharide injection; NEG CTRL, untreated muscle.

As shown in Figure 2, at 3 h (day 0) from the chemical and/or electrical stimuli no alteration of muscle morphology was observed. On day 7 after rHyal-*sk* plus GET administration, a modest effect on muscle morphology and a mild infiltrate of inflammatory cells were observed in the muscles for rHyal-*sk* molecule injected in combination with GET. This effect was comparable to that observed in bHyal treated muscles. On day 14 after rHyal-*sk* plus GET, inflammatory cells still appeared numerous in the muscles. Very similar results were obtained in the reference bHyal plus GET-treated muscles (Figure 2). The combination of Hyal plus GET showed a higher number of monocytes/macrophages in comparison to Hyal treatments alone demonstrating the role of GET in monocytes/macrophages recruitments. 

### 2.4. rHyal-sk Enhances the Inflammatory Cells Recruitment in Muscle after GET

**Figure 3:**
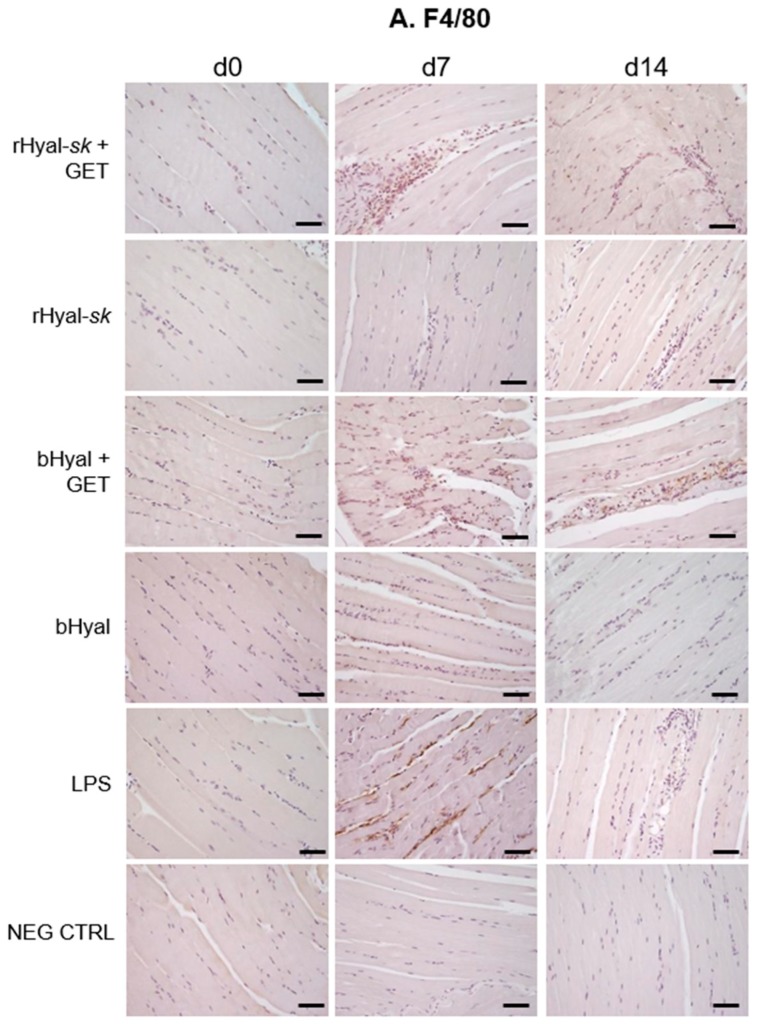

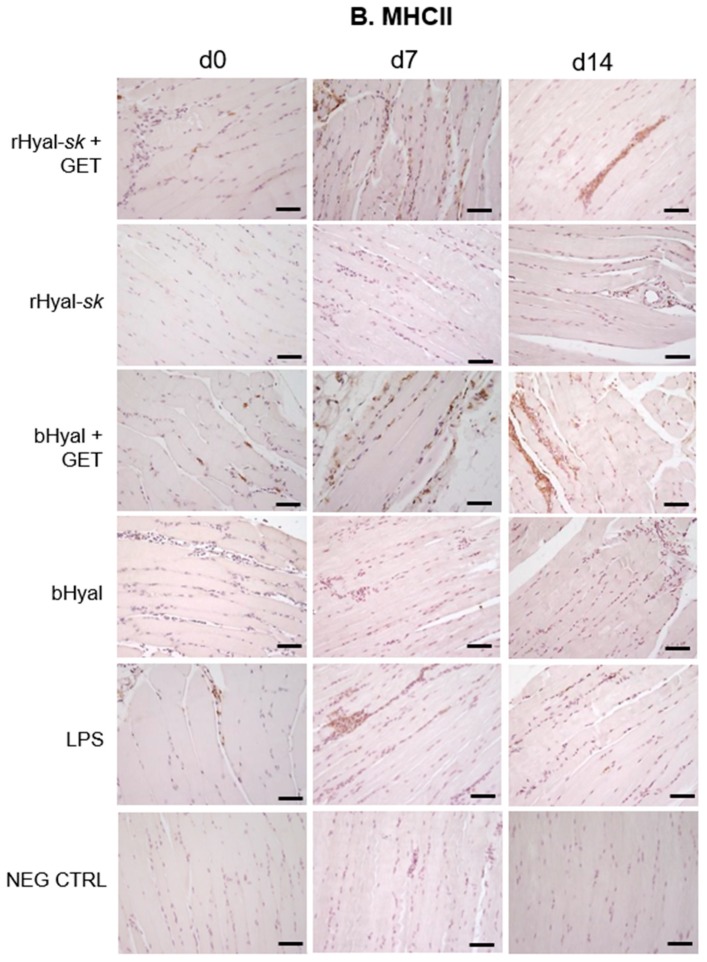

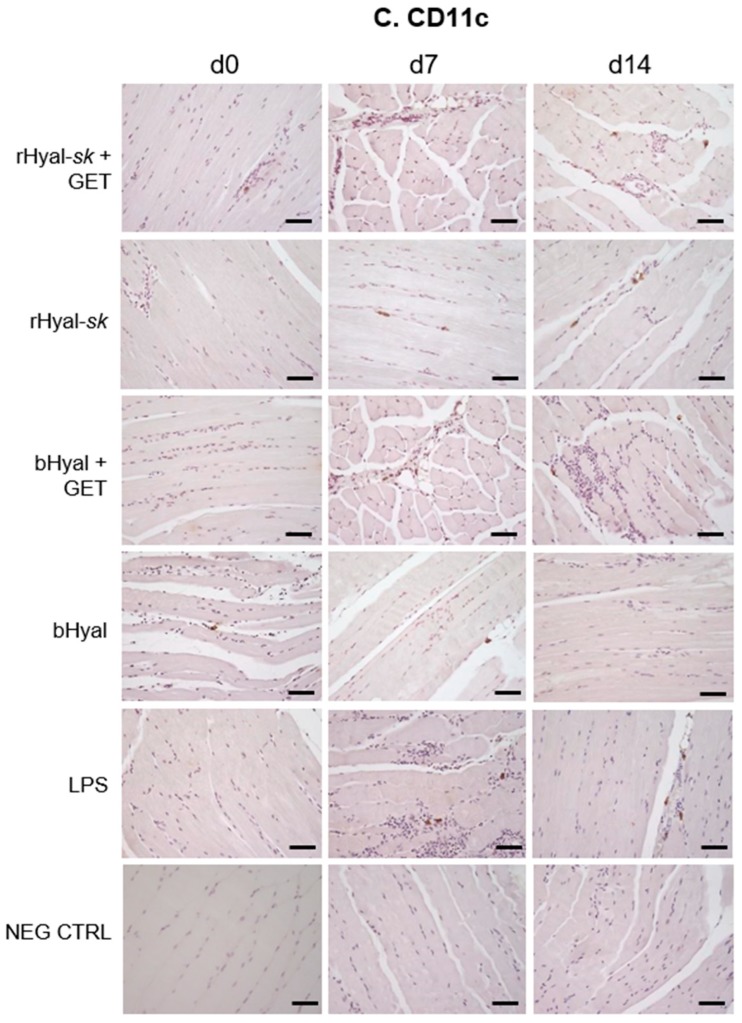
Immunochemistry of muscles treated with rHyal-*sk* in the presence and absence of GET. Skeletal muscle FFPE sections were stained at 3h (d0), d7 and d14 after treatment with monoclonal antibodies specific for macrophages (**A**, F4/80) and dendritic cells (**B**, MHC-II; **C**, CD11c). Positive control samples were muscles treated with LPS. Negative control samples included skeletal muscle harvested from untreated mice. All sections were counterstained with hematoxylin and analysed by optical microscopy. 20× magnification. Scale bars, 50 μm. rHyal-*sk*, recombinant Hyal from Fidia Farmaceutici; bHyal, bovine Hyal from Sigma-Aldrich; Hyal + GET, electrotransfer after Hyal injection; LPS, lipopolysaccharide injection; NEG CTRL, untreated muscle.

Injuries caused by the electrical field enroll a great quantity of monocytes/macrophages, which take part in muscle regeneration [45]. Therefore, we characterized the phenotype of the infiltrating cells recruited by rHyal-*sk* injection with or without GET at 3 h (d0), d7 and d14 after treatment with monoclonal antibodies specific for macrophages, focusing on F4/80+ macrophages and MHC-II+ and CD11c+ APCs. At these time points, the injection of rHyal-*sk* in absence of GET was never able to recruit a significant number of the three cell populations (Figure 3). 

GET coupled with rHyal-*sk*, induced a peak at d7 in the number of macrophages (F4/80-positive cells) and specialized APCs, which included macrophages and dendritic cells (MHC-II and CD11c-positive cells) similarly to the LPS treatment. Particularly, the number of CD11c-positive cell recruited at d7 appeared minor compared to the other cells (Figure 3). We showed that macrophage and APC migration to the inflammatory site was an early event, paralleling the results reported by Chiarella et al., who demonstrated that these cell populations were recruited on the site of treatment as soon as day 4 [21]. Here we observed that at day 7 residual cells still persisted. A decreasing intensity staining for F4/80 and CD11c surface markers was observed on d14 (Figure 3A,C). Nevertheless, a residual positivity to MHC-II staining was still detectable at d14 (Figure 3B). We obtained comparable results in the reference bHyal plus GET-treated muscles (Figure 3). 

### 2.5. No Production of Anti-rHyal-sk Antibodies after One Intramuscular Injection

**Figure 4:**
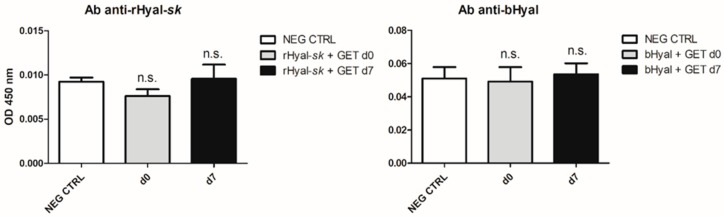
Anti-rHyal-*sk* antibody production. ELISA was performed with mouse sera (1:100 dilution) harvested 3 h (d0) (n = 4) and 7 days (d7) (n = 4) after Hyal injection in the presence of GET. No significant difference was observed between OD 450 nm of rHyal-*sk* + GET treated muscle d7 versus NEG CTRL, *p =* 0.84 (n.s.), equally between bHyal + GET treated muscle d7 versus NEG CTRL, *p =* 0.79 (n.s.). NEG CTRL, negative control (untreated muscle); rHyal-*sk*, recombinant Hyal, Fidia Farmaceutici; bHyal, bovine Hyal, Sigma-Aldrich; n.s., not significant. Statistical analysis: unpaired Student’s *t* test. Data are Mean ± SD.

We investigated the immune response of rHyal-*sk* when injected intramuscularly. Antibody levels against Hyal were measured in the serum of mice 7 days after the treatment with the enzyme injection associated to GET. It is known that the electrical stimulation, usually applied 1–2 h after the injection, does not modify the enzyme, maintaining unaltered its function, and that GET does not increase the physiological antibody response to Hyal [21]. Day 7 was selected for the high number of APCs recruited in the treated muscles at this time point (Figure 3), suggesting a possibly good presentation of Hyal by the migrated APCs to the resident lymphocytes. As shown in Figure 4, we observed the absence of antibody titer against rHyal-*sk* at day 7. Similarly, it was not observed any statistically significant immune response in electrotransferred bHyal-treated mice with respect to untreated mice.

### 2.6. rHyal-sk Plus GET Stimulates the Secretion of Proinflammatory Cytokines

It was already shown that electrotransfer with bHyal induces production of inflammatory cytokines in the first hours after treatment [21,44]. Here we analysed the profile of cytokines involved in inflammation, tissue injury and repair, specifically IL-1β, IL-6 and TNF-α, which were characteristic of acute inflammation. 

**Figure 5:**
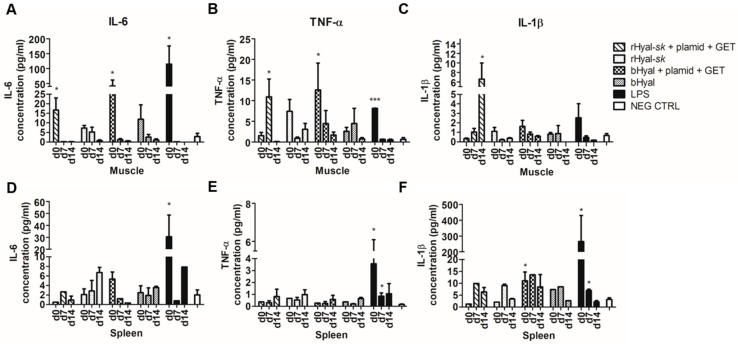
Measurement of IL-6, IL-1β and TNF-α levels in skeletal muscle and spleen of mice treated with rHyal-*sk* in the presence and absence of GET. Comparison of cytokines levels measured by ELISA. (**A**) IL-6 muscle: rHyal-*sk* + plasmid + GET day 0 versus Neg Ctrl, *p =* 0.0407 (*); rHyal-*sk* day 0 versus Neg Ctrl, *p =* 0.0584; bHyal + plasmid + GET day 0 versus Neg Ctrl, *p =* 0.05 (*); LPS d0 versus Neg Ctrl, *p =* 0.0404 (*). (**D**) IL-6 spleen: LPS d0 versus Neg Ctrl, *p =* 0.033 (*). (**B**) TNF-α muscle: rHyal-*sk* + plasmid + GET day 7 versus Neg Ctrl, *p =* 0.0387 (*); bHyal + plasmid + GET day 0 versus Neg Ctrl, *p =* 0.05 (*); LPS d0 versus Neg Ctrl, *p =* 0.0008 (***). (**E**) TNF-α spleen: LPS d0 versus Neg Ctrl, *p =* 0.0456 (*); LPS d7 versus Neg Ctrl, *p =* 0.0165 (*). (**C**) IL-1β muscle: rHyal-*sk* + plasmid + GET day 14 versus Neg Ctrl, *p =* 0.048 (*); bHyal + plasmid + GET day 0 versus Neg Ctrl, *p =* 0.066; LPS d0 versus Neg Ctrl, *p =* 0.0609. (**F**) IL-1β spleen: bHyal + plasmid + GET day 0 versus Neg Ctrl, *p =* 0.034 (*); LPS d0 versus Neg Ctrl, *p =* 0.0297 (*); LPS d7 versus Neg Ctrl, *p =* 0.0248 (*). rHyal-*sk*, recombinant Hyal from Fidia Farmaceutici; bHyal, bovine Hyal from Sigma-Aldrich; Hyal + plasmid + GET, electrotransfer after plasmid and Hyal injection; GET, electrogene transfer; LPS, lipopolysaccharide injection; NEG CTRL, negative control. Statistical analysis: unpaired Student’s *t* test.

Cytokines level measurement was performed at 3 h (d0), 7 days (d7) and 14 days (d14) after treatments (Figure 5). The earliest time point of 3 h was included to evaluate the inflammation-related cytokine release usually described as an early event occurring within 8 h after the application of a proinflammatory stress [21,44,46]. Then, the second time point at day 7, corresponded to the peak of the immune response, assuming that it was the best intermediate time point for detecting a peak of cytokine release. Day 14 was selected as the third time point to measure any possible late cytokines release.

Maximal IL-6 release in muscles was observed 3 h after rHyal-*sk* plus GET treatment (*p* = 0.0407) (Figure 5A). Its production dropped down to the detection limit at day 7 and day 14. TNF-α release in muscles was significantly increased 7 days after rHyal-*sk* plus GET treatment (*p* = 0.0387) (Figure 5B), but no cytokine production was measured in spleen after rHyal-*sk* plus GET treatment or rHyal-*sk* injection (Figure 5E). However, high levels of IL-1β in skeletal muscle were observed after 14 days rHyal-*sk* plus GET treatment (*p* = 0.0387), demonstrating a late release of this cytokine after GET coupled rHyal-*sk* administration differently from the effect of bHyal plus GET treatment (Figure 5C). The positive control, LPS, induced a significant secretion in muscles as well as in the spleen of IL-6 (muscle d0, *p* = 0.0404; spleen d0, *p* = 0.033), TNF-α (muscle d0, *p* = 0.0008; spleen d0, *p* = 0.0456) and IL-1β (muscle d0, *p* = 0.0297; spleen d0, *p* = 0.0248) 3h after treatment (Figure 5). As already demonstrated for the hyaluronidase of bovine extraction origin [21], no significant difference in cytokines production was observed in the spleen, indicating that there was no influence of the rHyal-*sk* treatment on this organ (Figure 5D–F).

Beside the difference in the timing of TNF-α and IL-1β maximal release in muscles between rHyal-*sk* + GET or bHyal + GET use (Figure 5B,C), no salient differences were observed between the use of rHyal-*sk* or bHyal on the production of IL-6 in muscles (Figure 5A) and spleens (Figure 5D). For both Hyals, we observed an early rise in IL-6 production 3 h after Hyal plus GET treatment in skeletal muscle, whereas no cytokine release was registered when Hyals were administered without GET.

## 3. Discussion

Several strategies have been proposed to potentiate DNA immunotherapy effectiveness in anti-cancer protocols, being plasmid DNA vaccines characterized by low immunogenicity. Chemical or physical devices have been exploited to increase DNA delivery into target organs, as the promising Gene-electro-transfer (GET) method.

Here we investigated a new kind of soluble recombinant bacterial Hyal, the rHyal-*sk*, in order to assess the effect of this Hyal treatment at the cellular and biochemical levels, when injected on skeletal muscle as adjuvant of GET-based immunotherapy protocols. The rHyal-*sk* is a recombinant Hyal that has a novel bacterial catalytic domain with high enzymatic activity [42]. Compared to commercially available animal-derived hyaluronidase, rHyal-*sk* is more thermostable [9,42] and presents higher proteolytic resistance, with activity over a broad pH spectrum [9,42]. In addition, it exhibits remarkable substrate specificity for HA [43] and no risk of animal cross-infection.

In this study, we evaluated whether rHyal-*sk* treatment influenced the efficiency of gene uptake in the muscular fibres and how the inflammatory process was activated by the administration of GET after rHyal-*sk* pretreatment. We assessed the cellular events and the inflammation chemical mediators involved in the first two weeks after rHyal-*sk* administration with and without the combination with GET. 

The comparative analysis of skeletal muscles morphology at three time points (3 h, 7 days, 14 days) revealed a slight tissue damage and recruitment of infiltrating monocytes and granulocytes in the early phase (7 days) after rHyal-*sk* pretreatment plus GET (Figure 2), which create a proinflammatory context useful in local antigen presentation in case of DNA vaccination protocols. The immunohistochemistry analysis (Figure 3) demonstrated indeed that the combination of rHyal-*sk* with GET favours the migration of high number of polymorphonuclear cells and APCs in comparison to rHyal-*sk* alone administration. As expected, the rHyal-*sk* did not induce any proinflammatory context in the absence of GET, similarly to the bHyal.

As shown in Figure 3, GET in presence of rHyal-*sk* pretreatment induced a visible infiltration of inflammatory cells 7 days after treatment. Additionally, we observed a clear cell recruitment into the muscle, mostly represented by F4/80+ macrophages and dendritic cells expressing MHC-II. A high number of such cell subpopulations into the damaged muscles was observed both at 7 and 14 days. This process was strictly linked to the substantial muscle regeneration observed at 14 days after the treatment with rHyal-*sk* with GET (Figure 2 and Figure 3), characterized by cell proliferation and differentiation of new myogenic cells that fused to existing damaged fibres for repair. When fusion of myogenic cells was completed, the size of the newly formed myofibres increased and myonuclei moved to the periphery of the fibre [44]. These data were in line with previous experimental observations obtained with a different type of Hyal of extraction origin [21,43,47] and implied that rHyal-*sk* could be used in optimized DNA immunotherapy protocols performed with GET using 14 days as the appropriate interval for DNA administration into skeletal muscle. This time is indeed required to create the proinflammatory environment necessary for APC recruitment and the myofibres regeneration fundamental for the uptake of DNA plasmid [48]. 

The analysis of the cytokine released upon treatment revealed an IL-6 production in muscles as early as 3 h after treatment with rHyal-*sk* plus GET, demonstrating the ability of such treatment to stimulate an early cytokine production (Figure 5). IL-6 production in muscles dropped down to the detection limit at day 7. No differences were observed between the use of rHyal-*sk* or bHyal on the production of IL-6. Conversely, a different timing of TNF-α and IL-1β production was observed in muscles treated with rHyal-*sk* and bHyal. TNF-α and IL-1β release in rHyal-*sk* plus GET treated muscles peaked at day 7 and day 14, respectively, and 3 h after treatment on bHyal plus GET treated muscles suggesting a long lasting inflammatory response induction by the rHyal-*sk* with respect to bHyal (Figure 5). In addition, we observed that bHyal plus GET induced a rapid production of TNF-α in the first hours after treatment while rHyal-*sk* plus GET induced a delayed pic of production 7 days after treatment. In view of these differences of TNF-α production kinetics, one could hypothesize that bHyal plus GET acts mainly on the innate immune response with a production mainly by macrophage and monocytes while the rHyal-*sk* plus GET activates the adaptive immune response and trigger a TNF-α production from lymphocytes.

Moreover, TNF-α and IL-1β production patterns for rHyal-*sk* plus GET and bHyal plus GET were quite different in the muscle where and when the GET is delivered (local effect). For both cytokines, the expression was high at day 0 with the bHyal pretreatment and then decreases. A different pattern was observed with the rHyal-*sk*. A low level was detected at day 0 followed by a sharp increase in the following days. A final decrease is present at day 14 for the TNF-α. This is not the case with IL-1β where a high level was observed at day 14.

We should consider that the endotoxin is a major contributing factor in inflammation in Hyals partially purified from bovine testis (bHyal) [49,50]. In addition, these preparations showed the presence of other proteins, which also may contribute to their proinflammatory activity [50]. Therefore, we could hypothesize that the only differences observed regard TNF-αand IL-1β production patterns for rHyal-*sk* plus GET and bHyal plus GET, could be due to the presence of endotoxin and some other components in protein preparation of bHyal, which stimulated the inflammatory response in vivo. In contrast, rHyal-*sk* is a hightly purified recombinant protein preparation, in fact it is free of pg amounts of endotoxin and other components contamination.

Apart from this, the treatment with rHyal-*sk* was able to activate an adequate response which could efficiently recruit APCs and macrophages in the site of injection for a suitable immunotherapy strategy. Importantly, cytokines release was not affected by rHyal-*sk* alone.

Since rHyal-*sk* is a Hyal obtained by a recombinant pathway in the non-pathogenic microorganism S. koganeiensis, it has low homology with the human enzyme. Consequently, we evaluated the level of its immunogenicity in vivo. We found that when repeatedly administered subcutaneously in rats, the presence of nADA could be detected following the highest dose and remains measurable for at least one month afterward. We also investigated the immune response of rHyal-*sk* when injected intramuscularly associated to GET and we did not observe any antibody titer against rHyal-*sk* at day 7 (Figure 4). More importantly, no particular toxicological findings were recorded in any of the treated animals. Therefore, while the induction of nADA could limit the use of rHyal-*sk* in treatments requiring repeated administrations for long periods, this enzyme can find use as an adjuvant to potentiate the immune response of DNA electrotransfer, which needs a single injection, and in all the “single treatments” into the clinics. 

Taken as a whole, our results indicate that rHyal-*sk* injection in skeletal muscles with the application of electric field pulses (1) induces a slight tissue damage and a recruitment of infiltrating monocytes and granulocytes, which produce a proinflammatory context useful for local antigen presentation in anti tumoral vaccination protocols; (2) is effective in terms of safety, in-take ability, immune response activation; (3) does not induce a significant production of nADA when injected once intramuscularly and associated to GET.

In view of these results, we support the adoption of rHyal-*sk* as a new constituent of immunotherapy protocols based on plasmid delivery, to potentiate the administration of DNA electrotransfer in the field of novel veterinary and human anti-cancer therapeutic strategies.

## 4. Materials and Methods 

### 4.1. Production of rHyal-sk, a Soluble Recombinant Bacterial Hyal

rHyal-*sk* (Fidia Farmaceutici, Local Unit Fidia Research Sud, Siracusa, Italy) was cloned through an expression system leading to the production of the bacterial Hyal soluble domain (aminoacids 31–247), which was identified and isolated from the *Streptomyces koganeiensis* DNA. The expression system was based on a pET vector, carrying the kanamycin resistance as selectable marker, the T7 expression region, the f1 origin and containing a gene construct encoding the soluble form of bacterial Hyal. rHyal-*sk* sequence data were submitted to GenBank under the accession number: KP313606. Protein 3D structure was deposited in EMBLEBI, Protein Data Bank (http://www.ebi.ac.uk/pdbe/) under accession number: PDB ID 4UFQ.

The *Escherichia coli* BL21 (DE3) competent cells were transformed with the selected vector. Following the fermentation process, the enzyme activity in the periplasmic extract from the bacterial culture was analyzed as briefly described: a pellet was produced from 100 μL of culture after centrifugation at 7500 rpm for 10 min. The cellular pellet was again suspended in 500 μL of B-PER Bacterial Protein Extraction Reagent (Pierce, Waltham, MA, USA), vortexed and centrifuged at 13,000 rpm for 10 min in order to separate the soluble proteins from the insoluble. By means of the turbidimetric assay [51,52], the collected supernatant was tested for the presence of Hyal activity. 

Subsequently, for the production of a suitable quantity of the soluble rHyal-*sk* destined specifically for this study, the recombinant bacterial Hyal was purified from the pellet via periplasmic extraction from osmotic shock and ultimately through three chromatographic steps: anion exchange (quaternary ammonium group, GE Healthcare, Chicago, IL, USA), cation exchange (carboxymethyl group, GE Healthcare), and hydrophobic interaction (phenyl sepharose, GE Healthcare). This purification sequence was followed by the analysis of purity and specificity through SDS-PAGE (12%) and enzymatic activity [51,52].

Contaminants of the *E. coli* were analyzed by ELISA for the host protein (Cygnus Technologies, Inc., Southport, NC, USA), by Threshold System for the host DNA, and by Chromo-LAL Kinetic Chromogenic Endotoxin Testing for the endotoxin content. Enzyme potency in bulk preparations was determined by the turbidimetric assay [51,52] using Ph. Eur. bovine Hyal as the reference standard. 

### 4.2. Toxicology Study and Evaluation of Anti-Drug Antibodies (ADA) Production

A 28-day toxicity study of rHyal-*sk* was performed in rats at Harlan Laboratories S.A. (Santa Perpetua de Mogoda, Barcelona, Spain). All animal procedures were executed in accordance with institutional guidelines for laboratory animal care, in adherence with ethical standards, and in compliance with good laboratory procedures (GLP). One hundred Wistar rats were distributed into four dose groups, each containing ten animals per sex, except for the control group (group 1) and the high dose group treated with the test item Hyal (rHyal-*sk*) at 10,500 U/kg (group 4), which contained a further five animals per sex for an additional 2-week treatment-free recovery period. The animals received the test item Hyal (rHyal-*sk*) subcutaneously every forty-eight hours for 4 weeks at the doses of 150 U/kg (Group 2), 1050 U/kg (Group 3) and 10,500 U/kg (Group 4). Control animals from Group 1 received the vehicle only, PBS, following the same regimen as the groups treated with rHyal-*sk*. At the end of the treatment period, all animals from the main study were necropsied and examined postmortem. The rest of the animals remained on-study, untreated, for additional 2 weeks and were necropsied and examined postmortem at the end of the recovery period. The injection sites and the following standard list of tissues were collected, processed for histology and evaluated by a veterinary pathologist: epididymides, esophagus, eyes with optic nerve, femur, heart, large and small intestine, kidneys, larynx, liver, lungs, lymph nodes (mandibular and mesenteric), lymph nodes that drain or are in contact with the injection sites (axillary and brachial), mammary gland area, ovaries, pancreas, peyer’s patches, pituitary gland, prostate and seminal vesicles, salivary glands, sciatic nerve, skeletal muscle, skin (abdominal), spinal cord, spleen, sternum (with bone marrow), stomach, testes, thymus, thyroid, tongue, trachea, urinary bladder, uterus, cervix, oviducts, vagina, and all gross lesions.

The following toxicological examination were performed in all the necroscopsied mice: mortality, histopathology, macroscopic and microscopic findings on the injection site and all the tissues listed above.

To check if there was a clear indication of an immunogenic potential of rHyal-*sk*, all samples which were confirmed positive for the presence of anti-drug antibodies (ADA) were further investigated in the neutralizing antibody (nAbs) assay. Samples that showed results above the respective run-specific screening Cut Point for nAbs were considered potentially positive and were further investigated in the confirmatory assay by immunocompetition for nAbs. 

The determination of nAbs against rHyal-*sk* was conducted in 3 male and 3 female rats treated subcutaneously every forty-eight hours for a period of 4 weeks (for a total of 14 administrations): group 1 (Control)—0 U/kg (only vehicle); group 2—150 U/kg rHyal-*sk*; group 3—1050 U/kg rHyal-*sk*; group 4—10,500 U/kg rHyal-*sk*. ADAs were determined from samples before administration on day 1 (acclimatization), at the end of the treatment period (week 4) and two weeks after treatment (recovery). This analytical phase was conducted at Harlan Laboratories Ltd., Itingen, BL, Switzerland, under GLP-compliant conditions. The determination of nAbs against rHyal-*sk* in serum samples was carried out with an enzymatic assay based on the Hyaluronidase ELISA assay kit (K-6000, Echelon Biosciences, Salt Lake City, UT, USA) developed and validated by Harlan Laboratories Ltd., in order to quantify Hyal activity inhibition due to binding antibodies in rat serum (5% rat serum matrix). Quality Controls (QCs) at concentrations of 0.05 µg/mL Low Quality Controls (LQC) and 1 µg/mL High Quality Controls (HQC) were used. The acceptance criteria were fully met as optical density (OD) of HQC > LQC > Negative Controls (NC) and ODs ≥ Screening Cut Point (SCP) were obtained for the QCs of the screening assay. The used NCs met the acceptance criteria of OD < SCP. For the confirmatory assay, the immunocompetition of HQC and LQC was investigated. For all immunocompeted QCs, the immunocompetition was higher than the Confirmatory Cut Point (CCP) of 79.1% (determined during validation).

### 4.3. Animal Treatment with rHyal-sk Plus GET

All experiments involving 36 six-weeks-old female BALB/c mice (Charles River Laboratories International, Inc. Wilmington, MA, USA) were approved by the Ethical Committee at the University Cattolica Sacro Cuore-Roma, Italy, according to the decree Prot. pdc. CESA/A/61/2011; experimental Protocol 48 G. Mice were equally distributed in 6 groups: (1) rHyal-*sk* plus GET, (2) bHyal plus GET, (3) rHyal-*sk* alone, (4) bHyal alone, (5) LPS, (6) untreated. 

In both groups 2 and 4 (reference groups) mice were injected with a bovine Hyal (bHyal) (H-4272, Sigma-Aldrich, St. Louis, MO, USA).

Ten units of rHyal-*sk* (for groups 1 and 3) or bHyal (for groups 2 and 4) were suspended in 30 µL of 0.15 M sodium phosphate buffer and intramuscularly injected in the gastrocnemius muscle of both posterior hind limbs 2 h prior other treatments, as performed in the study of McMahon et al. [18].

For GET treatment, mice were shaved after anaesthesia with a mixture of ketamine-medetomidine (Domitor) injected in the muscle of the anterior limb, just before the GET treatment. Mice previously treated with rHyal-*sk* (6 mice) or bHyal (6 mice) were intramuscularly injected with pCMV-CpGfree-tdTomato (Invivogen, Toulouse, France), a 4400 bp plasmid encoding the fluorescent protein TdTomato used as a reporter of GET efficiency. An amount of 25 µg of plasmid suspended in a final volume of 25 µL of 0.15 M sodium phosphate buffer was injected in the gastrocnemius muscle of both posterior limbs. The GET was done using tweezer electrodes with terminal circular plates, applied on the gastrocnemius muscle after conductive gel application. The electrical parameters consisted of 10 square-wave pulses of 175 V to cm (voltage to electrode gap), duration 20 ms, delivered at a frequency of 1 Hz using an ECM 830 generator (Harvard Apparatus, BTX Instrument Division, Holliston, MA, USA). Electrical parameters have been decided based on previous experiments aimed to optimize the GET conditions [21,44,53]. As a control of endotoxin induced inflammation, an additional group of 6 mice were injected intramuscularly with 25 µg of LPS (Sigma-Aldrich) in a volume of 25 µL. Negative controls were six untreated mice, injected only with PBS. Two mice per group were sacrificed by CO_2_ asphyxiation at the following time points: 3 h (d0), 7 days (d7) and 14 days (d14) after treatment and muscles of both legs and spleens were collected and divided for tdTomato fluorescence quantification, histological examination and dosage of cytokines secretion by ELISA.

### 4.4. Handling of Tissue Specimens and Histological Examination

Skeletal muscles and spleens were harvested from animals treated with rHyal-*sk* or bHyal alone or in combination with GET, immediately frozen in dry ice and stored at −80 °C or fixed in 4% paraformaldehyde for 24 h and embedded in paraffin. Some of the frozen organs were embedded in cryostat embedding medium (Bioptica, Milan, Italy) for cryosectioning, and the remaining tissues were used for protein extraction. Histological sections of formalin-fixed paraffin-embedded (FFPE) samples were prepared using routine procedures for histopathological analyses.

Briefly, serial sections of 4 μm thickness were cut, stained with hematoxylin and eosin (Bio-Optica, Milano, Italy), dehydrated and mounted using Eukitt (O Kindler GmbH & Co., Freiburg, Germany) and air-dried before morphological analysis.

### 4.5. Immunohistochemistry

Immunohistochemistry was performed on 4-μm-thick FFPE tissue sections after antigen retrieval in sodium citrate buffer (10 mM sodium citrate, pH 6), for 40 min at 95 °C.

Sections were incubated in a humid chamber overnight at 4 °C with the following primary antibodies used at 1:50 dilution in blocking buffer: anti-mouse F4/80 clone BM8, anti-mouse MHC class II clone M5/114.15.2, and anti-mouse CD11c, clone N418 (eBioscience, San Diego, CA, USA). Next, slides were incubated with a biotin-labelled secondary antibody and HRP-conjugated avidin for 30 min at room temperature. Detection was achieved using a substrate/chromogen mixture (DAB) and haematoxylin counterstaining. Incubation with the primary antibody was omitted for the negative controls. The immunostained slides were observed under an optical microscope (Leitz DMR; Leica, Wetzlar, Germany), 20× magnification, and the image data were analysed using NIS FreeWare 2.10 software (Nikon, Minato, Tokyo, Japan).

### 4.6. tdTomato Fluorescence Analysis

Skeletal muscles were fixed in 4% buffered formalin for 24 h and dehydrated with increasing scale of sucrose. Samples were embedded in cryostat-embedding medium (Bio-Optica, Milan, Italy) and frozen. Cryosections of 5 µm were cut with a cryostat (Leica), fixed with 4% paraformaldehyde, dried and rinsed in PBS for 10 min. Slides were stained with DAPI and mounted in Mowiol mounting medium. Microscopy was carried out using an upright Macroscopic-fluorescence microscope (Macroscope, Leica Microsystems SA, Rueil-Malmaison, France), equipped with a Cool Snap HQ Camera (Roper Scientific, Photometrics, Tucson, AZ, USA). Slides were imaged by fluorescence using appropriate filters (TdTomato: excitation filter, BP: 560/40 nm, emission filter 630/75 nm and autofluorescence: excitation filter, BP: 480/40 nm, emission filter, BP 527/30 nm). The files were stored with image acquisition software (Metavue, Metamorph, Molecular Devices, Sunnyvale, CA, USA). The files were stitched with the Adobe Photoshop software to obtain a single image of the whole muscle. Muscle area, mean and integrated fluorescence intensity were calculated with an analysis software (Image J) [54]. After a background correction, by applying a suitable threshold on the TdTomato images, TdTomato positive area, mean and integrated fluorescence intensity were measured in the limit of this threshold. Similarly, the entire muscle area was measured in the limit of a suitable threshold defined on the autofluorescence image.

### 4.7. Protein Extraction

Skeletal muscles and spleens were frozen in dry ice and stored at −80 °C. Protein mass of samples was extracted as previously described [53]. Briefly, organ samples were weighted, and 50 µL of lysis buffer (0.1% Igepal, 1 mM PMSF, 1 × Protease Cocktail inhibitor (Sigma-Aldrich)) were added for 10 mg of tissue. Muscle and spleen samples were homogenized using an electrical homogenizer (Ultraturrax IKA, Staufen Germany). After an incubation of 20 min on ice, suspensions were centrifuged 15 min at 20,000× *g* at 4 °C. Supernatants were collected, aliquoted and stored at −20 °C. Protein concentration was determined by the Qubit according to the instructions of the manufacturer (Invitrogen/Life Technologies, Carlsbad, CA, USA).

### 4.8. Measurement of Antibody Response to rHyal-sk Plus GET

Serum samples were collected from the tail vein of mice treated with rHyal-*sk* plus GET and mice treated with bHyal plus GET at 3 h (d0) and 7 days after the treatment. Serum samples were harvested from untreated mice as negative control. Nunc Maxisorp 96 well-plates (Nunc A/S, Roskilde, Denmark) were coated with rHyal-*sk* or bHyal (8 μg/mL) in 0.1 M NaHCO_3_ buffer, pH 9.6. Serum ELISA was performed as previously described [21].

### 4.9. ELISA for Cytokines Quantification

The concentration of IL-1β, IL-6 and TNF-α in the protein extracts was quantified by ELISA according to the instructions of the manufacturers (Invitrogen/Life Technologies and eBioscience). Briefly, plates were coated overnight at 4 °C with a capture antibody and blocked with a blocking buffer (3% BSA in PBS or diluent buffer) for 1 h at room temperature (RT). Plates were then incubated 2 h at RT with 50 μg of protein extract per well diluted in diluent buffer. After three washes in PBS-Tween20-0.5%, plates were incubated 1 h at RT with biotinylated detection antibody, washed, and incubated 30 min at RT with Avidin-Horseradish Peroxidase (HRP). After the last wash, plates were developed with 3,3′,5,5′-tetremethylbenzidine (TMB) substrate for 15–20 min. The reaction was stopped by adding 2 N H_2_SO_4_. The optical density was measured at 450 nm.

### 4.10. Statistical Analysis

Statistical analysis was performed using Prism 5 statistical software (GraphPad Software Inc., San Diego, CA, USA). Results were reported as the mean ± (standard deviation) SD of quadruplicates. Comparison of the ELISA results obtained in the different experimental groups was performed with the unpaired Student’s *t* test. Statistical analysis of the TdTomato fluorescence evaluation was performed using a Mann Whitney test. Differences with *p* value < 0.05 were considered statistically significant.

## 5. Conclusions

rHyal-*sk*, ensures higher purity levels and the absence of risks related with the transmission of viral material compared to the native Hyal of extraction origin. Here, we have demonstrated the efficacy and safety of this remarkable and innovative protein, in terms of slight tissue damage, good plasmid in-take ability, useful inflammatory response activation, and low immunogenicity, when used as an adjuvant to potentiate the immune response of DNA electrotransfer.

For the demonstrated efficacy of this innovative molecule, we are confident it will be possible to propose rHyal-*sk* for a wider use in the clinical practice and to move faster to more effective DNA-based cancer therapies in humans.

## Figures and Tables

**Table 1:** ADA induction experiment.

Group	Time Point	ADA Positive Samples/Total Samples	ADA Positive Animals/Total Animals
Group 1: 0 U/Kg Control (Vehicle)	Before administration d1	0/6	0/6
	At the end of the treatment period	0/6	
	At the end of the recovery period	0/6	
Group 2: 150 U/kg rHyal-*sk*	Before administration d1	0/6	6/6
	At the end of the treatment period	6/6	
	At the end of the recovery period	6/6	
Group 3: 1050 U/kg rHyal-*sk*	Before administration d1	0/6	5/6
	At the end of the treatment period	5/6	
	At the end of the recovery period	5/6	
Group 4: 10,500 U/kg rHyal-*sk*	Before administration d1	0/6	2/6
	At the end of the treatment period	2/6	
	At the end of the recovery period	2/6	

ADAs were determined from samples before administration on day 1 (acclimatization), at the end of the treatment period (week 4) and two weeks after treatment (recovery). Determination was carried out based on an ELISA method validated in Harlan Laboratories (Santa Perpetua de Mogoda, Barcelona, Spain).

## References

[1] Chiarella P., Fazio V.M., Signori E. (2013). Electroporation in DNA vaccination protocols against cancer. Curr. Drug Metab..

[2] Jedrzejas M.J., Stern R. (2005). Structures of vertebrate hyaluronidases and their unique enzymatic mechanism of hydrolysis. Proteins.

[3] Stern R., Jedrzejas M.J. (2006). Hyaluronidases: Their genomics, structures, and mechanisms of action. Chem. Rev..

[4] Foley M.J., Wood W.B. (1959). Studies on the pathogenicity of group A Streptococci. II. The antiphagocytic effects of the M protein and the capsular gel. J. Exp. Med..

[5] Dale J.B., Washburn R.G., Marques M.B., Wessels M.R. (1996). Hyaluronate capsule and surface M protein in resistance to opsonization of group A streptococci. Infect. Immun..

[6] Petrey A.C., de la Motte C.A. (2014). Hyaluronan, a crucial regulator of inflammation. Front. Immunol..

[7] Bookbinder L.H., Hofer A., Haller M.F., Zepeda M.L., Keller G.A., Lim J.E., Edgington T.S., Shepard H.M., Patton J.S., Frost G.I. (2006). A recombinant human enzyme for enhanced interstitial transport of therapeutics. J. Control. Release.

[8] Wiegand R., Brown J. (2010). Hyaluronidase for the management of dextrose extravasation. Am. J. Emerg. Med..

[9] Messina L., Musumeci L., Vaccaro S. (2014). Fidia Farmaceutici, S.P.A. Bacterial Hyaluronidase and Process for Its Production. Patent.

[10] Amphadase™ (hyaluronidase) Injection Amphastar Pharmaceuticals, Inc.–DA Application No.: 021665. Approval Date: 10/16/2004. https://www.accessdata.fda.gov/drugsatfda_docs/nda/2004/21-665_Amphadase.cfm.

[11] Hydase™ (hyaluronidase) Injection PrimaPharm, Inc.–FDA Application No.: 021716. Approval Date: 10/25/2005. https://www.accessdata.fda.gov/drugsatfda_docs/nda/2005/021716s000_HydaseTOC.cfm.

[12] Vitrase™ (hyaluronidase) Injection ISTA Pharmaceuticals, Inc.–FDA Application No.: 021640. Approval Date: 5/5/2004. https://www.accessdata.fda.gov/drugsatfda_docs/nda/2004/21-640_Vitrase.cfm.

[13] Hylenex™ (hyaluronidase) Human Injection Halozyme Therapeutics, Inc.–FDA Application No.: 021859. Approval Date: 12/02/2005. https://www.accessdata.fda.gov/drugsatfda_docs/nda/2005/021859_s000_HylenexTOC.cfm.

[14] Bergqvist S. (1950). Increased reaction to BCG vaccination caused by hyaluronidase. Nord. Med..

[15] Salvioli G., Degli Esposti A., Dina M.A. (1952). Experimental studies on Salvioli’s diffusing vaccine; study of histobiochemical reactions in vaccination with killed Koch’s bacilli with and without hyaluronidase. Boll. Ist. Sieroter. Milan..

[16] Tozuka T., Niimura A., Yajima T., Takeda H. (1954). The enhancement of the effect of B.C.G. vaccination with hyaluronidase. J. Shinshu Univ..

[17] Wanisch K., Kovac S., Schorge S. (2013). Tackling obstacles for gene therapy targeting neurons: Disrupting perineural nets with hyaluronidase improves transduction. PLoS ONE.

[18] McMahon J.M., Signori E., Wells K.E., Fazio V.M., Wells D.J. (2001). Optimisation of electrotransfer of plasmid into skeletal muscle by pretreatment with hyaluronidase–increased expression with reduced muscle damage. Gene Ther..

[19] Vandermeulen G., Daugimont L., Richiardi H., Vanderhaeghen M.L., Lecouturier N., Ucakar B., Préat V. (2009). Effect of tape stripping and adjuvants on immune response after intradermal DNA electroporation. Pharm. Res..

[20] Cemazar M., Golzio M., Sersa G., Escoffre J.M., Coer A., Vidic S., Teissie J. (2012). Hyaluronidase and collagenase increase the transfection efficiency of gene electrotransfer in various murine tumors. Hum. Gene Ther..

[21] Chiarella P., De Santis S., Fazio V.M., Signori E. (2013). Hyaluronidase contributes to early inflammatory events induced by electrotransfer in mouse skeletal muscle. Hum. Gene Ther..

[22] Neumann E., Schaefer-Ridder M., Wang Y., Hofschneider P.H. (1982). Gene transfer into mouse lyoma cells by electroporation in high electric fields. EMBO J..

[23] Teissie J., Golzio M., Rols M.P. (2005). Mechanisms of cell membrane electropermeabilization: A minireview of our present (lack of ?) knowledge. Biochim. Biophys. Acta.

[24] Aihara H., Miyazaki J. (1998). Gene transfer into muscle by electroporation in vivo. Nat. Biotechnol..

[25] Heller R., Jaroszeski M., Atkin A., Moradpour D., Gilbert R., Wands J., Nicolau C. (1996). In vivo gene electroinjection and expression in rat liver. FEBS Lett..

[26] Muramatsu T., Shibata O., Ryoki S., Ohmori Y., Okumura J. (1997). Foreign gene expression in the mouse testis by localized in vivo gene transfer. Biochem. Biophys. Res. Commun..

[27] Rols M.P., Delteil C., Golzio M., Dumond P., Cros S., Teissie J. (1998). In vivo electrically mediated protein and gene transfer in murine melanoma. Nat. Biotechnol..

[28] Heller R., Heller L.C. (2015). Gene electrotransfer clinical trials. Adv. Genet..

[29] Daud A.I., DeConti R.C., Andrews S., Urbas P., Riker A.I., Sondak V.K., Munster P.N., Sullivan D.M., Ugen K.E., Messina J.L. (2008). Phase I trial of interleukin-12 plasmid electroporation in patients with metastatic melanoma. J. Clin. Oncol..

[30] Chiarella P., Massi E., De Robertis M., Fazio V.M., Signori E. (2008). Strategies for Effective Naked-DNA Vaccination Against Infectious Diseases. Recent Pat. Antiinfect. Drug Discov..

[31] Bagarazzi M.L., Yan J., Morrow M.P., Shen X., Parker R.L., Lee J.C., Giffear M., Pankhong P., Khan A.S., Broderick K.E. (2012). Immunotherapy against HPV16/18 generates potent TH1 and cytotoxic cellular immune responses. Sci. Transl. Med..

[32] Lee S.H., Danishmalik S.N., Sin J.I. (2015). DNA vaccines, electroporation and their applications in cancer treatment. Hum. Vaccine Immunother..

[33] Cemazar M., Golzio M., Sersa G., Rols M.P., Teissié J. (2006). Electrically-assisted nucleic acids delivery to tissues in vivo: Where do we stand?. Curr. Pharm. Des..

[34] Olaiz N., Signori E., Maglietti F., Soba A., Suárez C., Turjanski P., Michinski S., Marshall G. (2014). Tissue damage modeling in gene electrotransfer: The role of pH. Bioelectrochemistry.

[35] Keiser H.D., Hatcher V.B. (1979). The effect of contaminant proteases in testicular hyaluronidase preparations on the immunological properties of bovine nasal cartilage proteoglycan. Connect. Tissue Res..

[36] Williams R.G. (1955). The effects of continuous local injection of hyaluronidase on skin and subcutaneous tissue in rats. Anat. Rec..

[37] Eberhart A.H., Weiler C.R., Erie J.C. (2004). Angioedema related to the use of hyaluronidase in cataract surgery. Am. J. Ophthalmol..

[38] Silverstein S.M., Greenbaum S., Stern R. (2012). Hyaluronidase in ophthalmology. J. Appl. Res..

[39] Frost G.I. (2007). Recombinant human hyaluronidase (rHuPH20): An enabling platform for subcutaneous drug and fluid administration. Expert Opin. Drug Deliv..

[40] Baxter (2018). Annex 1, Summary of Product Characteristics (Hyqvia). AIFA. https://farmaci.agenziafarmaco.gov.it/aifa/servlet/PdfDownloadServlet?pdfFileName=footer_003822_042804_RCP.pdf&retry=0&sys=m0b1l3.

[41] Zacks Equity Research (2012). Bad News for ViroPharma. Yahoo Finance. https://finance.yahoo.com/news/bad-news-viropharma-211440512.html.

[42] Messina L., Gavira J.A., Pernagallo S., Unciti-Broceta J.D., Sanchez Martin R.M., Diaz-Mochon J.J., Vaccaro S., Conejero-Muriel M., Pineda-Molina E., Caruso S. (2016). Identification and characterization of a bacterial hyaluronidase and its production in recombinant form. FEBS Lett..

[43] Pavan M., Beninatto R., Galesso D., Panfilo S., Vaccaro S., Messina L., Guarise C. (2016). A new potential spreading factor: Streptomyces koganeiensis hyaluronidase. A comparative study with bovine testes hyaluronidase and recombinant human hyaluronidase of the HA degradation in ECM. Biochim. Biophys. Acta.

[44] Chiarella P., Massi E., De Robertis M., Sibilio A., Parrella P., Fazio V.M., Signori E. (2008). Electroporation of skeletal muscle induces danger signal release and antigen-presenting cell recruitment independently of DNA vaccine administration. Expert Opin. Biol. Ther..

[45] Karalaki M., Fili S., Philippou A., Koutsilieris M. (2009). Muscle regeneration: Cellular and molecular events. In Vivo.

[46] Zhou H.R., Islam Z., Pestka J.J. (2003). Kinetics of lipopolysaccharide-induced transcription factor activation/inactivation and relation to proinflammatory gene expression in the murine spleen. Toxicol. Appl. Pharmacol..

[47] Arnold L., Henry A., Poron F., Baba-Amer Y., van Rooijen N., Plonquet A., Gherardi R.K., Chazaud B. (2007). Inflammatory monocytes recruited after skeletal muscle injury switch into antiinflammatory macrophages to support myogenesis. J. Exp. Med..

[48] Peng B., Zhao Y., Lu H., Pang W., Xu Y. (2005). In vivo plasmid DNA electroporation resulted in transfection of satellite cells and lasting transgene expression in regenerated muscle fibres. Biochem. Biophys. Res. Commun..

[49] Dong Y., Arif A., Olsson M., Cali V., Hardman B., Dosanjh M., Lauer M., Midura R.J., Hascall V.C., Brown K.L. (2016). Endotoxin free hyaluronan and hyaluronan fragments do not stimulate TNF-α, interleukin-12 or upregulate co-stimulatory molecules in dendritic cells or macrophages. Sci. Rep..

[50] Huang Z., Zhao C., Chen Y., Cowell J.A., Wei G., Kultti A., Huang L., Thompson C.B., Rosengren S., Frost G.I. (2014). Recombinant human hyaluronidase PH20 does not stimulate an acute inflammatory response and inhibits lipopolysaccharide-induced neutrophil recruitment in the air pouch model of inflammation. J. Immunol..

[51] Di Ferrante N. (1956). Turbidimetric measurement of acid mucopolysaccharides and hyaluronidase activity. J. Biol. Chem..

[52] Hofinger E.S., Spickenreither M., Oschmann J., Bernhardt G., Rudolph R., Buschauer A. (2007). Recombinant human hyaluronidase Hyal-1: Insect cells versus *E. coli* as expression system and identification of low molecular weight inhibitors. Glycobiology.

[53] Chiarella P., Signori E. (2014). Intramuscular DNA vaccination protocols mediated by electric fields. Methods Mol. Biol..

[54] Rasband W.S. (1997–2016). ImageJ.

